# Ovarian relapse in a child with B-ALL: a case report

**DOI:** 10.11604/pamj.2025.51.50.45756

**Published:** 2025-06-18

**Authors:** Meriem Cheikhna, Chaimae El Mahdaoui, Nisrine Bennani Guebessi, Asmaa Zeggwagh, Nisrine Khoubila, Mouna Lamchahab, Meryem Qachouh, Mohamed Rachid, Abdellah Madani, Siham Cherkaoui

**Affiliations:** 1 Hematology and Pediatric Oncology Department of August 20 Hospital, Ibn Rochd University Hospital, Casablanca, Morocco,; 2 Laboratory of Cellular and Molecular Pathology, Faculty of Medicine and Pharmacy, Hassan II University of Casablanca, Morocco,; 3 Pathology laboratory, Ibn Rochd University Hospital, Casablanca, Morocco,; 4 Hda Laboratories of Medical Biology Analysis, Casablanca, Morocco

**Keywords:** Acute lymphoblastic leukemia, ovarian, B-ALL, case report

## Abstract

This case presents an 8-year-old girl diagnosed with B-cell acute lymphoblastic leukemia (B-ALL), who relapsed after 3 years of treatment and 1 year of complete remission, with an unusual extramedullary relapse in the ovary. Ovarian relapse of B-ALL is extremely rare in children, making this case noteworthy in scientific literature. The patient had an initial diagnosis of B-ALL with a deletion of chromosome 12, a genetic alteration previously associated with the ETV6-RUNX1 fusion gene, which is typically linked to a favorable prognosis but also carries a 20% risk of late relapse. The relapse was initially asymptomatic and went undetected until clinical symptoms of pelvic pain appeared. Imaging with pelvic ultrasound confirmed the ovarian involvement. The relapse was treated with standard chemotherapy protocols for B-ALL, resulting in a partial response. This case underscores the importance of considering extramedullary relapse in the differential diagnosis for pediatric ALL patients who present with atypical symptoms after remission. It also suggests that routine pelvic ultrasound could be a useful tool for early detection of ovarian and other extramedullary relapses, which are often associated with bone marrow relapse. The main take-away from this case is the necessity for vigilant follow-up, including targeted imaging, in ALL patient's post-remission to ensure early identification of extramedullary relapses, which can otherwise be easily overlooked. The presence of chromosome 12 deletion and its association with late relapse highlights the need for ongoing surveillance even in patients with initial favorable genetic abnormalities.

## Introduction

Acute lymphoblastic leukemia stands as the most prevalent form of childhood cancer, occurring in approximately 80% of children aged 1 to 10 years [1]. Treatment and outcomes of Acute lymphoblastic leukemia have improved dramatically in the past 10 years. Yet, relapsed ALL is considered one of the leading causes of death in childhood cancers and is estimated at 15% of cases in Acute lymphoblastic leukemia [2]. In cases of relapse in pediatric acute lymphoblastic leukemia (ALL), the bone marrow, central nervous system, and testes are widely recognized as common sites. On the other hand, ovarian relapse is rare, and its occurrence has decreased with the recent advancements in chemotherapy [3]. When a child with a confirmed diagnosis of leukemia experiences ovarian torsion, it is crucial to consider the possibility of disease relapse involving leukemia infiltration of the ovary, which may contribute to the occurrence of torsion [4].

## Patient and observation

**Patient Information:** an 8-year-old female patient arrived at the Pediatric Hematology and Oncology Department of 20 August Hospital Casablanca. She had a previous diagnosis of acute lymphoblastic leukemia (precursor B-cell subtype), established in 2018, and managed with standard risk chemotherapy. The patient was not hyper leukocytic, without infiltration of the central nervous system and corticosteroid sensitive. Following successful treatment, she was in complete remission and undergoing regular monitoring.

**Clinical findings:** in 2022, she presented with a painful abdominal swelling that was gradually enlarging, occurring alongside a decline in her overall health. Clinical examination revealed a palpable hard pelvic mass measuring 11 cm (about 4.33 in), with no other associated signs. Complete blood count showed pancytopenia, and abdominal ultrasound revealed an intraperitoneal abdominal-pelvic mass appearance.

### Diagnostic assessment

**Figure 1:**
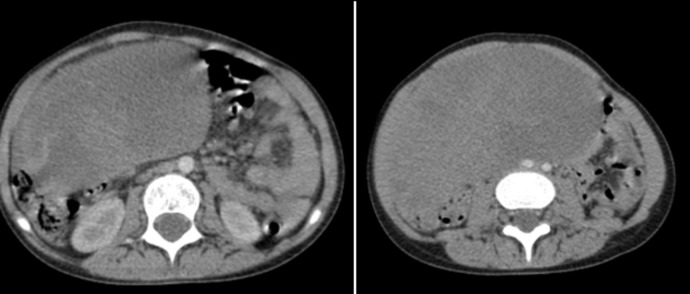
the axial section of a pelvic computed tomography scan showing a right ovarian mass

**Abdominopelvic computed tomography scan:** the abdominal-pelvic swelling has been progressively increasing in size over the past 20 days (about 3 weeks), with ultrasound revealing an intraperitoneal abdominal-pelvic mass. A well-defined oval-shaped intra-peritoneal abdominal mass was observed, heterogeneous in density, with heterogeneous enhancement after contrast injection, showing multiple areas of necrosis, measuring 144 x 74 mm (about 2.91 in) and extending over 158 mm (about 6.22 in) (Figure 1).

**Figure 2:**
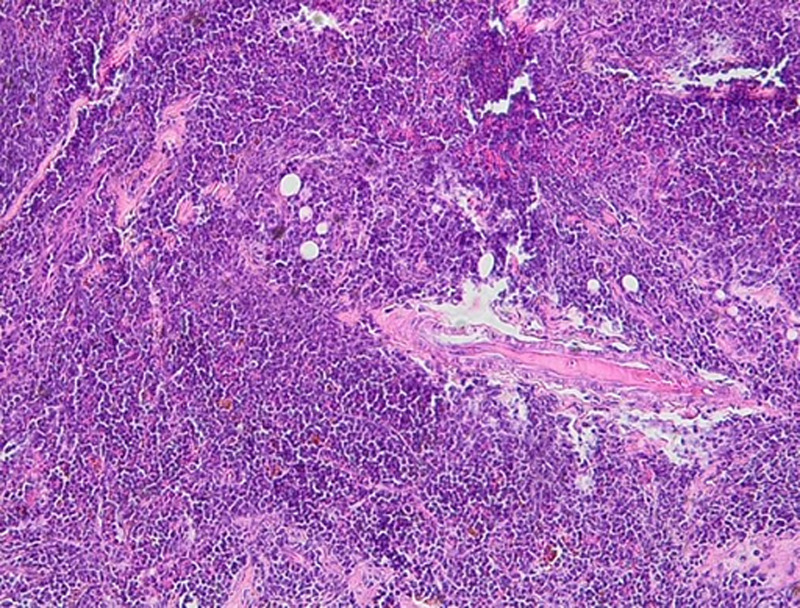
blastic lymphoid tumor proliferation with diffuse architecture

**Figure 3:**
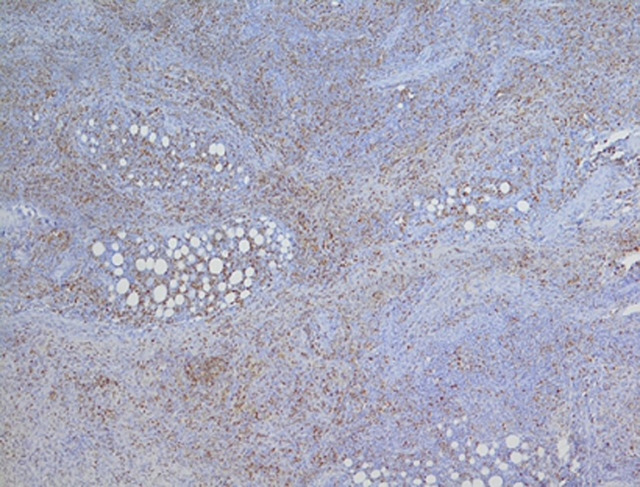
immuno-histochemistry using anti-Tdt antibody was positive, confirming ovarian relapse

**Ovarian histology:** a right salpingo-oophorectomy was performed. The histological examination concerns a biopsy fragment originating from a fibroadipose tissue, occupied by a dense lymphoid infiltrate with a diffuse or vaguely nodular architecture. The infiltrate consists of round cells of small to medium size, monomorphic, with finely granular chromatin and scanty cytoplasm. Histological examination revealed a dense lymphoid infiltration (Figure 2). Immunohistochemical analysis showed diffuse expression of Tdt-positive tumor cells and heterogeneous expression of CD20. CD3 was detected in reactive small T lymphocytes (Figure 3).

**Myelogram:** myelogram showed the presence of 90 to 95% blast cells, consistent with a medullary relapse of B-cell acute lymphoblastic leukemia (B-ALL). The diagnosis of ovarian and medullary relapse was established.

**Cytogenetic analysis:** her karyotype after relapse was presented as the following 45, XX, -1, der (3) (add (3) (q12), -5, -6, del (12) (p1;2), +mar1, +mar2 (2)/46, idem, +mar3|21/46, XX (2). Showing an initial hypodiploid clone with 45 chromosomes with complex clonal abnormalities in number and structure including: a derivative of chromosome 3 by loss and addition of material of unknown origin in 9q12, deletion of the short arm of chromosome 12, monosomies for chromosomes 1, 5, and 6, and 2 marker chromosomes of unknown origin (2 metaphases). A subclone with an additional third marker chromosome (2 metaphases). A major diploid clone without detected anomalies under the examination conditions (22 metaphases). The post relapse karyotype presented new abnormalities compared to the diagnostic karyotype, indicating clonal evolution. Her initial Karyotype revealed a minor clone with a translocation (3;5) and deletion of 12p (3 mitoses) The translocation (3;5) (q21;931) is typically reported in myeloid proliferations, 46,XX, t(3;5)(921;931), del(12) (p11p13) (31/46, XX (31).

**Therapeutic interventions and follow-up:** the patient was admitted to the National Marall Standard Risk protocol but was switched to the Frall protocol upon relapse.

**Patient perspective:** after a long period of remission, the patient and her family were initially distressed by the recurrence of symptoms and the discovery of a pelvic mass. The diagnosis of ovarian and medullary relapse came as a shock, but it also brought clarity to the cause of her discomfort. Despite the emotional toll of resuming intensive treatment, the patient remained cooperative and resilient throughout the therapeutic process. Her parents expressed relief at having a clear diagnosis and appreciated the medical team´s commitment to preserving her long-term health. The family remains hopeful and engaged, grateful for the close monitoring and follow-up that allowed early detection and management of the relapse.

**Informed consent:** written informed consent was obtained from the patient for publication of this case report and any accompanying images. A copy of the written consent is available for review by the Editor-in-Chief of this journal.

## Discussion

Patients diagnosed with acute lymphoblastic leukemia (ALL) may experience relapse in sanctuary sites such as the brain, ovaries, or testes, even if the bone marrow shows signs of remission [5]. Ovarian torsion, a medical emergency that can affect individuals of all ages, occurs when the vascular pedicle of the ovary undergoes complete or partial rotation, resulting in compromised blood flow. In 50-90% of cases, it may be linked to an underlying ovarian lesion [4]. Recurrence in the bone marrow is the most prevalent, observed in 50%-60% of instances. Approximately 20% of cases involve relapse in the central nervous system, around 5% in testicular disease, with ovarian relapse being infrequently recorded [6], but with the advance in science through years in ALL research some studies clarify and report more about case reports related to ovarian relapse [4,7-9]. The occurrence of ovarian masses as a manifestation of ALL recurrence remains infrequent. While ovarian relapse is not commonly observed in females with ALL, its incidence may rise as patient survival rates improve with combination chemotherapy [10].

## Conclusion

Relapses of B-cell acute lymphoblastic leukemia (B-ALL) in the ovaries are exceptionally uncommon in pediatric cases, often presenting insidiously and remaining undetected until symptoms arise. Timely detection is crucial as ovarian relapse is frequently linked to relapse in the bone marrow or other sites outside the bone marrow. Regular pelvic ultrasound examinations are advised for early detection of extramedullary relapses, particularly those in the pelvic region.
